# Structural racism through Sundown towns and its relationship to COVID-19 local risk and racial and ethnic diversity

**DOI:** 10.1016/j.pmedr.2023.102260

**Published:** 2023-05-24

**Authors:** Yulin Hswen, Franklin Yang, Circe Le-Compte, Margaret E. Hurley, Heather Mattie, Thu. T. Nguyen

**Affiliations:** a Department of Epidemiology and Biostatistics, University of California San Francisco, San Francisco, CA, USA; b Bakar Computational Health Sciences Institute, University of California San Francisco, San Francisco, CA, USA; c Department of Biostatistics, Harvard T.H Chan School of Public Health, Boston, MA, USA; d Department of Psychiatry and New York State Psychiatric Institute, Columbia University, New York, NY, USA; e University of Massachusetts of Amherst, Amherst, MA, USA; f University of Maryland School of Public Health, College Park, MD, USA

**Keywords:** Structural racism, Segregation, COVID-19, Racism, Race

## Abstract

“Sundown towns” across the US prevented racial and ethnic minorities from living and working within their borders as they forced minorities to leave these towns after sunset. The objective of this study was to explore the relationship between sundown town status, COVID-19 local risk index and racial and ethnic diversity. A multi-level hierarchical model was used to examine the effect of historical segregation through sundown towns status on present day COVID-19 local risk index and city-level diversity. Over 2,400 Sundown towns were cataloged across the United States, with the greatest density in the Midwest. Sundown towns, which historically excluded racial and ethnic minorities, had significantly less city-level diversity and lower COVID-19 local risk index compared to non-Sundown towns. Findings show that Sundown towns perpetuate residual segregation which continues to impact current inequities in COVID-19 risk among racial and ethnic minorities at the neighborhood level. We recommend that public health officials for pandemic preparedness should devote greater resources to these historically segregated racial and ethnic minority areas because of the historic structural racism that has placed these places at higher risk.

## 1 Introduction

“The slavery that survived long past emancipation was an offense permitted by the nation, perpetrated across an enormous region over many years and involving thousands of extraordinary characters.”

- Douglas A. Blackmon, Slavery by Another Name: The Re-Enslavement of Black Americans from the Civil War to World War II.

While the Emancipation Proclamation of January 1, 1863 ended slavery, it failed to ensure equitable access to social freedoms for Blacks in the U.S. As the final slaves in Galveston, Texas received their freedom, in June 19, 1865, thousands of cities across the U.S. quickly passed laws to build, reinforce, and sustain racial segregation and inequity among racial and ethnic minorities in the U.S. This special brand of jurisprudence ensured that the oppression of racial and ethnic minorities in the country did not end. In addition to Jim Crow laws, convict leasing, peonage, and involuntary servitude, cities passed “sundown town” ordinances that allowed racial and ethnic minorities to work in the city during the day, but required them to leave the city limits before nightfall (Loewen, 2005, Esquibel, 2011).

Some cities employed signage and slogans explicitly enforcing racial restrictions (Loewen, 2005):

“N----r, Don’t Let the Sun Go Down on You in Alix.”

-Alix, Arkansas^1^

“A Good Place to Live…No Negroes.”

-Edmond, Oklahoma^1^

“Cool Summers, Mild Winters, No Blizzards, No Negroes.”

-Mena, Arkansas^1^

An example is the town of Anna, Illinois is infamously known for its long history of racial exclusion. The name of the town itself originates from the acronym A-N-N-A, which stands for “Ain’t No N----ers Allowed.” Towns like Anna are not exclusive, or uncommon (Loewen, 2005); starting around 1890 until 1968 (Loewen, 2005), people in the United States set up thousands of white towns across the country.

Sundown towns used psychological to physical forms of threats to uphold their exclusionary laws. These ranged from passive surveillance and intimidation to organized raids and racial cleansing. These tactics often were coupled with restrictive covenants, which limit how land can be used and by whom, (Esquibel, 2011) and freeze-outs, systematic denial of services in restaurants, motels, health care, and housing, to expel persons of color (Loewen, 2005, Beaujot, 2018 May).

Sundown towns also varied in size and affluence. Sizes ranged from small hamlets such as De Land, Illinois (500 inhabitants) to large cities such as Appleton, Wisconsin^1^ (57,000 inhabitants in 1970) (Loewen, 2005, Li and Yuan, 2022). Sundown towns were ubiquitous in the United States and racial segregation did not just exist deep in the south. Southern states only had relatively few Sundown towns because of the region’s pervasive Jim Crow laws, which greatly limited Black autonomy. The state of Mississippi, located in the country’s southern Gulf State region, has approximately six Sundown towns, while it has been estimated that the state of Illinois, had 472 Sundown Towns (Loewen, 2005). This growing peer-reviewed literature on Sundown Towns showcases that these were forms of local racial caste in American society (Lancaster, 2010, Givel, 2021, Bell, 2008).

### 1.1 Intergenerational neighborhood effects

Based on previous work by Sharkey and Elwert (2011), the neighborhood environment in one generation may have an impact on future generations. Neighborhood effects have often been studied as a static feature and ignore the neighborhood stratification of time (Sharkey and Elwert, 2011). However, more evidence by Sharkey and Elwert showed the negative effects of multigenerational neighborhood inequality across two consecutive generations while controlling for the effects of treatment and confounders over time. Thus, multigeneration disadvantages such as the persistence of poor neighborhood economic status are transmitted to multiple generations, with a parent’s childhood neighborhood environment influencing the next generations' childhood. Specifically, a parent’s childhood neighborhood exerts a lingering influence on a child’s cognitive ability through the resources that are available to the child which includes the home environment and schooling (Sharkey and Elwert, 2011).

More recently, evidence of the long-running effects of the 1930 s Home Owners Loan Corporation (HOLC) redlining maps - areas were “redlined” by lending institutions through the denial of investment that would improve housing and economic opportunity, whereby negative effects on labor market outcomes, family structure, and incarceration was seen on cohorts born several decades after the maps had been produced (Aaronson et al., 2021). Our study on the impact of Sundown towns on COVID-19 local risk draws from the literature on multigenerational neighborhood effects. While Sundown towns faded from popular consciousness at end of the twentieth century and following the banning of redlining under the Fair Housing Act of 1968, Sundown town segregation remained (Loewen, 2005, Esquibel, 2011). Most of these historic Sundown towns continue to have only small populations of people of color to this day, which may have reinforced explicit and subconscious biases among majority groups in these communities.

Even though Sundown towns are outlawed, the enduring legacy of racial segregation and structural racism may impact the health inequities experienced by racial and ethnic minority populations in these historic Sundown towns today. Indeed, spatial stratification by racial segregation can serve to maintain and produce inequality across multiple dimensions and generations over time (Faber and Sharkey, 2015). We have seen that the history of racialized exclusion from “redlining” from the Home Owners Loan Corporation (HOLC) subsequently has shaped persistent geographic segregation, contemporary housing inequality and geographic patterns of vulnerability.[11,12.13] Our research seeks to extend the literature and understand how Sundown towns, a method of structural segregation extends towards impacting the current day local COVID-risk.

The purpose of this study was to examine the relationship between historic sundown town status and the risk of COVID-19 during the 2020 pandemic (Latkin et al., 2021). COVID-19 has disproportionately impacted communities of color, judged through measures of both infections and deaths (APM Research Lab). Since the start of the pandemic, an inequitable distribution that has laid “bare inequalities and anxieties, discrimination and division.” (Leach et al., 2020) Compared to non-Hispanic whites, African-American/Black and Hispanic groups are approximately 3 times more likely to experience hospitalization, and 2 times more likely to die from COVID-19 (Centers for Disease Control and Prevention. Risk for COVID-19 infection, hospitalization, and death by race/ethnicity, 2021). Jim Crow laws in the U.S. often informed systematic abuses of racial and ethnic minorities by medical providers and researchers, creating enduring and understandable mistrust of the health care system (Bajaj and Stanford, 2021). Current research indicates that non-Hispanic people of color are less likely to trust vaccination efforts (Latkin et al., 2021, Raude et al., 2016 Jul 2, Laurencin, 2021 Jun, Jimenez et al., 2021). Understanding the impact of historic structural racism may help to identify communities at greater need for focused public health efforts today and in the future.

## 2 Methods

Our hypothesis was that cities with a history of Sundown towns in the United States would have decreased levels of racial and ethnic minority diversity and higher levels of COVID-19 local risk. Our secondary hypothesis is that a mediator between Sundown towns and COVID-19 local risk is city-level diversity, as these populations were excluded from Sundown towns and forced to confine to non-sundown town areas.

To investigate the effect of historic sundown town status on local COVID-19 local risk in over 500 cities across the U.S., we first identified cities that were Sundown towns and mapped each to its corresponding county. We then built a multi-level hierarchical model that explored the relationship between city-level COVID-19 local risk and city-level diversity, sundown town status, and county-level median income and poverty. We also conducted a mediation analysis to address and control for the relationship between COVID-19 local risk and city-level diversity.

### 2.1 Sundown towns

The (History and Social Justice) Justice website, inspired by Loewen’s work, created an online registry of Sundown towns across the United States based on U.S. census data, newspapers, local histories, and oral histories chronicling discriminatory customs and practices (History and Social Justice). The database designates one webpage per cataloged sundown town. Within each webpage, each town is classified with a status indicating what type of sundown town it was, depending on the amount of evidence indicating it was a sundown town: “Possible”, “Probable”, or “Surely”. Only towns with the most evidence of being Sundown towns, those labeled with a “Probable” or “Surely” status, were used in our analyses.

### 2.2 COVID-19 local risk score and Racial andEthnic diversity

Data compiled by the (City Health Dashboard) project, an initiative led by NYU Langone Health and sponsored by the Robert Wood Johnson Foundation, (City Health Dashboard) was used to quantify COVID-19 local risk score and racial/ethnic diversity. The (City Health Dashboard) project contains data for over 750 U.S. cities. Metrics range from health outcomes (breast cancer deaths, colorectal deaths, etc.) to socioeconomic factors (absenteeism, income inequality, diversity, etc.).

COVID-19 local risk measures estimated city-level risk of COVID-19 infection based on several social and environmental factors (City Health Dashboard). Values range from 1 to 10, where 1 indicates areas of lowest risk and 10 indicates areas of highest risk. COVID-19 local risk index is calculated based on chronic conditions and demographic characteristics that may increase the risk of complications from COVID-19.

Minority population and diversity measures drive a minor but important contribution to COVID-19 local risk index. The “Social Vulnerability” and “COVID-19-related Demographics” categories specifically have some components that are collinear minority-related covariates. These components collectively produce a 16% contribution to COVID-19 local risk: “Group 3: Minority Status & Language” under Social Vulnerability contributes 4%, and “Minority” under COVID-19-related Demographics contributes 12%. We’ve included covariates related to diversity and minority at the county and city-level to account for these contributions in an effort to specifically study the impact of sundown town status on COVID-19-risk.

Racial and ethnic diversity quantifies the mix of different racial and ethnic groups within a geographic area, with values ranging from 0 to 100. A score of 0 indicates that all citizens within the geographic area belong to one racial and ethnic group, while 100 indicates that all racial and ethnic groups are found in equal proportion (City Health Dashboard). A higher city-level diversity is associated with a higher percentage of racial and ethnic minorities at the county level. We selected the diversity index as a variable of interest because we hypothesize that Sundown Towns will have a lower diversity index because these towns excluded racial and ethnic minorities from staying past sundown.

### 2.3 Census data

We enriched the city-level data sources with census data by mapping each city to its corresponding county. For each city, we identified the county and Federal Information Processing Standard Publication (FIPS) county code; a five-digit code used to uniquely identify counties and county equivalents in the US (National Institutes of Standards and Technology). The FIPS codes were used to merge our city-level dataset with county-level covariates: the percentage of households under the federal poverty limit and median income (National Institutes of Standards and Technology). Then, each city was mapped to a geographical region: Northeast, South, West, and Midwest (National Institutes of Standards and Technology).

### 2.4 Statistical analysis

We used a hierarchical linear model with 3 levels to explore the relationship between COVID-19 local risk, racial/ethnic diversity, and historic sundown town status. We accounted for variations in city (level 1)*,* county (level 2) and region-level (level 3) contributions to local COVID-19 local risk by adding a random intercept for each in our mixed-effects model. This model was constructed as follows:

yijk = *βγijk* + *μ*0*i* + *e*0*ij* + *v*0*ijk*.

[*μ*0*j*] ∼ *N* (0,*σ*^2_*region*).

[*e0ij*] ∼ *N* (0,*σ*^2_*state*).

[*v0ijk*] ∼ *N* (0,*σ*^2_*city*).

where *yijk* represents the COVID-19 local risk score for each city; *β* represents the fitted model parameters which vary by model, but include sundown town status, demographics such as percentage of minorities, and geographic region;. The parameters *μ*0*i*, *e*0*ij*, and *v*0*ijk* represent the residuals for each level: region, county, and city respectively.

We also investigated the mediation effects of city-level diversity on sundown town status and COVID-19 local risk index. We hypothesized city-level diversity mediates the impact of sundown town status on COVID-19 local risk score since discriminatory traditions in Sundown towns would have an effect on overall levels of diversity today. We used Baron and Kenny’s method to analyze the mediation effect. First, we regressed COVID-19-risk scores against sundown town status. Then, we looked at the effect of sundown town status on city-level diversity. Finally, we regressed COVID-19-risk scores against sundown town status and city-level diversity.

## 3 Results

### 3.1 Sundown towns

A full list of sundown town statuses and corresponding counts is displayed in Table 1.

**Table 1:** Sundown town status and number of towns in each status category.

Status	Counts
Probable	737
Surely	360
Total	1097

A total of 2,479 Sundown towns were cataloged in the 48 contiguous U.S. Sundown towns were geocoded to a correspondent county or city based on the (History and Social Justice) Justice database. A total of 2,026 of the 2,469 Sundown towns could be mapped to US counties, with 1,097 of these cities categorized as “Probable” or “Surely” Sundown towns (See Table 1).

**Fig. 1:**
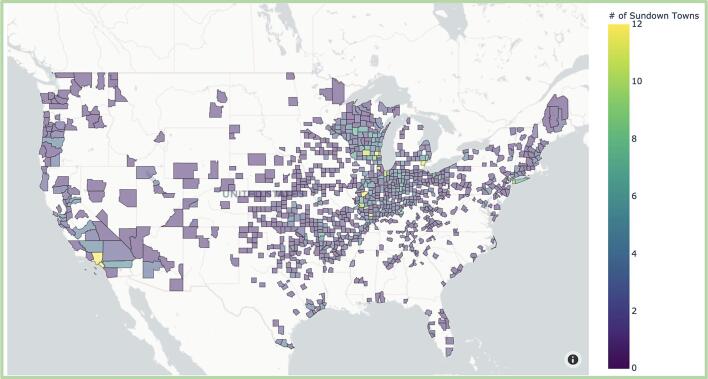
Geographic distribution of Sundown towns across the U.S.

A map of the total number of “Probably” and “Surely” Sundown towns per county across the U.S. is presented in Fig. 1. Table 2 shows the distribution of Sundown towns across the geographic regions in the US. Sundown towns are distributed across states with a mean of 23.8 and median of 8 total Sundown towns per state (25th and 75th percentile = [3.25, 21]). Counties with at least one sundown town had an average of 1.686 Sundown towns and a median of 1 sundown town (25th and 75th percentile = [1,2]).

**Table 2:** Distribution of Sundown towns across geographic regions of the United States.

Level	Mean	25% Percentile	Median	75% Percentile
County	1.686	1	1	2
State	23.849	3.25	8	21

Region	Number of Sundown town			
Midwest	644			
Northeast	55			
South	254			
West	144			

Overall, the clustering and volumes of Sundown towns were highest in the Midwest, as shown in Table 2 and Fig. 1. The Midwest had 644 Sundown towns, the South had 254, the West had 144, and the Northeast had 55. Few counties had more than 2 Sundown towns, while the Midwest had the highest number of counties with 10 or more Sundown towns.

The mediation effects of city-level diversity on Sundown town status and COVID-19 local risk index are presented in Table 3. When city-level diversity was regressed against Sundown town status, Sundown town status was found to have a significant negative effect on city-level diversity with a coefficient of −4.847 (p = 0.002). Sundown town status also had a significant negative relationship with COVID-19 local risk score, with a coefficient of −0.698 (p = 0.018). When COVID-19 local risk was regressed against both Sundown town status and city-level diversity, the effect of Sundown town status remained significant; however, the coefficient of sundown town status decreased to −0.680 (difference of 0.018, p = 0.022), indicating partial mediation.The estimated effect of city-level diversity is no longer significant (p = 0.585).

**Table 3:** Mediation effects of city-level diversity on sundown town status and COVID-19 local risk index.

	Diversity Index		COVID Risk Score		COVID Risk Score adjusting for city-level diversity	
Fixed Effects	Estimate	P Value	Estimate	P Value	Estimate	P Value
City is a Sundown Town	−4.847	0.002	−0.698	0.018	−0.680	0.022
Diversity					0.004	0.585

The effect of city-level diversity, county-level poverty levels, county-level median income, region, and Sundown town status on COVID-19 local risk score is presented in Table 4. Model 1 regresses COVID-19 local risk score against city-level diversity, county-level median income, county-level poverty levels, region and Sundown town status. City-level diversity, country-level median income, and region were statistically significant predictors. City-level diversity and a city being in the West region had positive coefficients of 0.018 (p = 0.004) and 1.682 (p = 0.002) respectively. Median income and Sundown town status were negatively associated with COVID-19 local risk score with coefficients of −0.817 (p < 0.001) and −1.169 (p = 0.009) respectively. Model 2 exchanges city-level diversity for percent minority as a predictor. Percent minority was found to be a significant predictor for COVID-19 local risk, with a coefficient of 2.428 (p = 0.010). Median income, Sundown town status, and being in the West region remained significant predictors, with coefficients of −0.862 (p < 0.001), −1.444 (p = 0.001), and 1.958 (p < 0.001) respectively. Notably, coefficients for Sundown town status and median income decreased from −1.169 and −0.817 respectively in Model 1 to −1.444 and −0.862 respectively in Model 2. Meanwhile, the coefficient for the West region increased from 1.682 to 1.958 between Model 1 and Model 2 respectively.

**Table 4:** Models exploring the effect of city-level and county-level covariates on COVID-19 local risk.

	Model 1 (Diversity and Perc Poverty and Sundown Town)		Model 2 (Minority and Perc Poverty and Sundown Town)	
Fixed Effects	Estimate	P Value	Estimate	P Value
Percent Minority			2.428	0.010
Diversity	0.018	0.004		
Median Income	−0.817	0.000	−0.862	0.000
Percent Poverty	0.040	0.288	0.012	0.770
City was a Sundown town	−1.169	0.009	−1.444	0.001
Region = Northeast	2.266	1.000	2.364	0.249
Region = South	0.401	1.000	0.328	0.872
Region = West	−0.365	1.000	−0.261	0.898
City was a Sundown town in the Northeast	−0.822	0.549	−0.497	0.717
City was a Sundown town in the South	0.069	0.930	0.404	0.608
City was a Sundown town in the West	1.682	0.002	1.958	0.000
Random Effects	Variance			
County-level variance	1.241		1.256	
Region-level variance	1.560		1.424	
Residual	1.978		1.975	

Model 3 (Diversity and Minority and Perc Poverty and Sundown Town)				
Fixed Effects	Estimate	P Value		
Percent Minority	1.743	0.077		
Diversity	0.014	0.032		
Median Income	−0.880	0.000		
Percent Poverty	0.014	0.738		
City was a Sundown Town	−1.259	0.005		
Region = Northeast	2.252	0.286		
Region = South	0.274	0.896		
Region = West	−0.319	0.879		
City was a Sundown Town in the Northeast	−0.683	0.619		
City was a Sundown Town in the South	0.207	0.794		
City was a Sundown Town in the West	1.783	0.001		
Random Effects				
County-level variance	1.244			
Region-level variance	1.467			
Residual	1.973			

Model 3 is a combination of Model 1 and Model 2, where both city-level diversity and percent minority are included as covariates. When both diversity and percent minority are included, percent minority is no longer significant (p = 0.077) and the coefficient of city-level diversity decreased slightly from 0.02 to 0.014. City-level diversity and being in the West region remain the only covariates positively correlated with COVID-19 local risk score, with coefficients of 0.014 (p = 0.032) and 1.783 (p = 0.001) respectively. Median income and Sundown town status were both negatively correlated with coefficients of −0.880 (p < 0.001) and −1.259 (p = 0.005) respectively.

Across all models, median income, Sundown town status, and the indicator for Sundown towns in the West stayed significant with the coefficients retaining their signs.

County-level variance, region-level variance, and residual terms were similar across all three models. In Model 1, county-level variance was 1.241, in Model 2 this increased to 1.256, and in Model 3 the county-level variance was 1.244. Region-level variance was 1.560 in Model 1 and increased to 1.424 in Model 2. In Model 3, region-level variance settled at 1.467, which was between the values in Model 1 and Model 2. Residual terms across all three models were similar: 1.978 in Model 1, 1.975 in Model 2, and 1.973 in Model 3.

## 4 Discussion and future directions

### 4.1 Principal findings

Our study shows a distinct relationship between historic Sundown town status and COVID-19 local risk among racial and ethnic minorities living in those areas. The results of our study indicate that cities of increased diversity and counties of lower median income have higher COVID-19 local risk index. Historic Sundown towns status had a negative association with COVID-19 local risk index and the city diversity index. In each model, median income also had a strong statistically significant relationship with risk scores. The stability of this finding, even with the introduction and removal of covariates, supports those more affluent areas are at lower risk for COVID-19 infection. Despite median income being a significant covariate in all models, the percentage of households in poverty was not, which could be attributed to possible collinearity between poverty and other covariates in our model, such as median income, city-level diversity, and percent minority population.

Unlike Jim Crow laws, which were primarily concentrated in the South, Sundown towns are distributed across the country with a median of 8 Sundown towns per state and the highest concentration of Sundown towns in the Midwest region. These findings are consistent with recent studies by the Brookings Institute which found that minority groups are poorly represented in Midwestern counties (Frey). Though the Sundown town laws have been abolished, they may have left ongoing legacies that inform social norms and environments impacting racial and ethnic minority groups to this day.

Sundown towns most likely were not as prevalent in the South due to the historic segregation minority populations in the region since the Colonia era, which were encoded in Jim Crow laws and systematic acts of terrorism following the end of slavery. Since segregation was sponsored at the state level, Sundown town practices were not necessary because these laws were practiced widely across the state. States with limited or no histories of slavery, smaller populations of racial and ethnic minorities, or lacking Jim Crow statutes, had higher volumes of Sundown towns. The largest cluster is found in the Midwest, outside the band of Jim Crow states.

A significant association between Sundown town status and diversity was found (−4.847, p = 0.002) with Sundown towns having a city-level diversity index of 4.850 points lower than non-Sundown towns. This indicates that historic Sundown towns have lower levels of diversity likely a residual effect of not allowing racial and ethnic minorities. There was an association with Sundown Towns and COVID-19 local risk index (−0.698, p = 0.18) whereby there was less risk for COVID-19 in Sundown towns. However, when Diversity index was included in this model that significance disappeared indicating that present racial and ethnic Diversity was a mediator for Sundown town status. These results indicate the segregation enforced by historic Sundown towns has persisted to present day and has impacted present day health risks such as COVID-19. Racial and ethnic minority populations in these towns continue to have limited social, economic, and health equity relative to the whites, as evidenced by their less optimal health outcomes for both infectious and non-communicable diseases and lowered lifespan (Molotch et al., 2000 Dec, Malina et al., 2021, Kihlström and Kirby, 2021 Jul). This effect of structural exclusion is further exacerbated by the impact of historic segregation on present-day attitudes, particularly among whites in Sundown towns, who may be unaware of historic Sundown town laws and practices and may believe that the absence of people of color today is a product of choice instead of a byproduct of historic segregation (Arrington, 2015, Best et al., 2021).

### 4.2 Relevance to previous studies

The positive association between risk scores and Sundown town status is consistent with other studies that have examined the persistence of negative neighborhood racialized segregation across multiple generations over time (Faber and Sharkey, 2015, Faber, 2017, Faber, 2020, O’Connell, 2019 Jul, Perez-Stable and Hooper, 2021 May 20, Schoen, 2018). Racial mortgage lending practices such as “redlining” have had significant long-term effects on the economic and social opportunity on cohorts born several decades after the abolishment of these inequitable mortgage practices (Aaronson et al., 2021). In epidemiological studies, it has been shown that Jim Crow laws have a statistically significant relationship to current health inequities, including higher rates of infant mortality (Schoen, 2018, Krieger et al., 2013 Dec) higher risk and poor prognoses for breast cancer, (Krieger et al., 2018) dementia, (Farina et al., 2018) diabetes, (Fleischer et al., 2016) heart disease (Kramer et al., 2017).

Based on our results, these neighborhood segregation practices of Sundown towns have translated into disparate COVID-19-related health outcomes as well, where people of color and minorities are disproportionately affected by COVID-19 infections and death (Iacobucci, 2020, Wang et al., 2020 Jun, Yearby, 2021 Mar 4). Increased social vulnerability scores are a predictor of a higher risk for COVID-19. Counties with a greater proportion of minority residents, particularly Black Hispanic residents, have had higher incidences of COVID-19 cases (Islam et al., 2021). These trends are mirrored in trajectories of COVID-19 vaccine intentions among U.S. adults. Racial and ethnic inequities have been closely tied to both enduring histories of medical abuse and vaccine hesitancy and vaccine uptake (Karpman et al., 2021, Strully et al., 2021 Apr). People of color have exhibited the lowest probability of likely getting vaccinated, and the gap between people of color and other racial groups has, in most cases, increased over time (Niño et al., 2021). These repeated patterns of health inequities disproportionately affecting minority groups are indicative of the country’s longstanding history of racial exclusion, displacement, and segregation (Aaronson et al., 2021, Faber and Sharkey, 2015, Faber, 2017, Faber, 2020, Sharkey and Faber, 2014, Momplaisir et al., 2021, Iacobucci, 2020).

### 4.3 Limitations

There are limitations on the registry of Sundown towns used in this study. This registry from the (History and Social Justice) Justice was compiled using a mix of many sources. A sundown town is defined by its repeated efforts to drive minorities away. However, these rules were not always codified into law like Jim Crow laws. Therefore, the registry of Sundown towns itself draws from both written and oral accounts. For instance, Loewen estimated that at “least 3,000 and perhaps as many as 15,000 independent towns, plus another 2,000 to 10,000 suburbs, could be classified as Sundown towns”.^1^ However, Loewen did not provide a list of these Sundown Towns or a database for this analysis. As, a result, there could be significantly more Sundown towns than those currently identified and our results could be an underestimation of its impacts.

It is important to note that this study is ecological in design and provides a correlational relationship between Sundown towns and COVID-19 outcomes and does not establish a causal relationship. Although we found a significant association between Sundown towns and COVID-19 local risk scores, which is a composite index that incorporates several aspects that contribute to how vulnerable specific cities are to the COVID-19 virus, and control for various sociodemographic and economic variables, there are other factors that we may not have accounted for that could have contributed to this association that is confounding the relationship.

### 4.4 Future directions

Our study builds upon the mounting evidence linking racial exclusion with negative health outcomes among minority groups. We examine this relationship on a national scale in the context of the COVID-19 pandemic. Future studies should build upon previous research showing that non-Hispanic people of color have decreased odds of COVID-19 vaccine trust compared to whites, (Hswen et al., 2020) and delve deeper into the potential association between Sundown town status and rate of COVID vaccination.

Additionally, exclusionary practices such as those exhibited by Sundown towns have been shown to have negative effects not only on minority populations, but communities as a whole. There is a clear association between the level of social capital and the level of health and safety within a community, which implies that combating systemic racism helps build healthier and safer communities that benefit everyone (Lancaster and Kirk, 2014). Future studies should address the lasting negative effects of historic Sundown towns on not only people of color, but also the current-day population living within the borders.

Ultimately, our research reiterates the multi-generation impact of racialized historical policies. The United States is structurally rooted in racist and exclusionary policies as a method to maintain a local racial caste society. Effective change in demographic inequities are dependent upon change targeting the source of the problems. At a legislative level, these changes must address issues of social inequity and provide minority groups with the resources to prevent and combat long term negative health outcomes.

### 4.5 Public health implications

Starting in the late 1800s, thousands of cities adopted Sundown laws, implementing spoken and unspoken rules to prevent racial and ethnic minorities from living and working within their borders for nearly a century. The impact of these laws, and the violence and discrimination encoded and reified through them, are felt to this day—particularly in the Midwest, where Sundown towns are most densely concentrated. Our findings indicate that towns documented with sure or probable Sundown status are negatively associated with current diversity levels and COVID-19 local risk index. This implies that past exclusionary policies have intergenerational consequences, particularly among people of color. Greater research to examine these intergenerational effects based on historical segregation is necessary to be able to further improve the health of those and the future generations that have been impacted.

Anti-discrimination policies that are structural and institutional are needed to prevent the perpetuation of racial and ethnic segregation. Future pandemic preparedness should include the assessment of historical structural racism to ensure communities that have been historically discriminated against obtain the necessary resources to reduce their risk of disease. Greater awareness of past segregation policies that have influenced risk in racial and ethnic communities should be part of public health planning.

## 5 Ethics

The study is considered that the analysis of de-identified, publicly available data does not constitute human subjects research as defined at 45 CFR 46.102 and that it does not require IRB review.

## Funding

Research reported in this publication was supported by the National Cancer Institute, under Contract No. 75N91020R00039 (YH) National Institute on Minority Health and Health Disparities R00MD012615 (TTN), R01MD015716 (TTN). The content is solely the responsibility of the authors and does not necessarily represent the official views of the National Institutes of Health.

## Declaration of Competing Interest

The authors declare that they have no known competing financial interests or personal relationships that could have appeared to influence the work reported in this paper.

## Data Availability

Data will be made available on request.
